# Early Prediction of Severe COVID-19 in Patients by a Novel Immune-Related Predictive Model

**DOI:** 10.1128/mSphere.00752-21

**Published:** 2021-10-13

**Authors:** Caiyu Sun, Mingshan Xue, Min Yang, Lihui Zhu, Yunxue Zhao, Xiaoting Lv, Yueke Lin, Dapeng Ma, Xuecheng Shen, Yeping Cheng, Haocheng Xuan, Xiaoqing Jia, Tao Li, Lihui Han

**Affiliations:** a Shandong Provincial Key Laboratory of Infection and Immunology, Shandong Provincial Clinical Research Center for Immune Diseases and Gout, Department of Immunology, School of Basic Medical Sciences, Cheeloo College of Medicine, Shandong University, Jinan, Shandong, China; b State Key Laboratory of Respiratory Disease, National Clinical Research Center for Respiratory Disease, Guangzhou Institute of Respiratory Health, First Affiliated Hospital of Guangzhou Medical University, Guangzhou, China; c Department of Gastroenterology, Shandong Provincial Hospital Affiliated to Shandong First Medical University, Jinan, Shandong, China; d Department of Pharmacology, School of Basic Medical Sciences, Cheeloo College of Medicine, Shandong University, Jinan, China; e Department of Gastroenterology, Qilu Hospital, Shandong University, Jinan, Shandong, China; f Department of Infectious Diseases, Shandong Provincial Hospital Affiliated to Shandong First Medical University, Jinan, Shandong, China; Mount Sinai School of Medicine

**Keywords:** COVID-19, IgA, SARS-CoV-2, eosinophils, neutrophils, predictive model

## Abstract

During the progression of coronavirus disease 2019 (COVID-19), immune response and inflammation reactions are dynamic events that develop rapidly and are associated with the severity of disease. Here, we aimed to develop a predictive model based on the immune and inflammatory response to discriminate patients with severe COVID-19. COVID-19 patients were enrolled, and their demographic and immune inflammatory reaction indicators were collected and analyzed. Logistic regression analysis was performed to identify the independent predictors, which were further used to construct a predictive model. The predictive performance of the model was evaluated by receiver operating characteristic curve, and optimal diagnostic threshold was calculated; these were further validated by 5-fold cross-validation and external validation. We screened three key indicators, including neutrophils, eosinophils, and IgA, for predicting severe COVID-19 and obtained a combined neutrophil, eosinophil, and IgA ratio (NEAR) model (NEU [10^9^/liter] − 150×EOS [10^9^/liter] + 3×IgA [g/liter]). NEAR achieved an area under the curve (AUC) of 0.961, and when a threshold of 9 was applied, the sensitivity and specificity of the predicting model were 100% and 88.89%, respectively. Thus, NEAR is an effective index for predicting the severity of COVID-19 and can be used as a powerful tool for clinicians to make better clinical decisions.

**IMPORTANCE** The immune inflammatory response changes rapidly with the progression of severe acute respiratory syndrome coronavirus 2 (SARS-CoV-2) infection and is responsible for clearance of the virus and further recovery from the infection. However, the intensified immune and inflammatory response in the development of the disease may lead to more serious and fatal consequences, which indicates that immune indicators have the potential to predict serious cases. Here, we identified both eosinophils and serum IgA as prognostic markers of COVID-19, which sheds light on new research directions and is worthy of further research in the scientific research field as well as clinical application. In this study, the combination of NEU count, EOS count, and IgA level was included in a new predictive model of the severity of COVID-19, which can be used as a powerful tool for better clinical decision-making.

## INTRODUCTION

Since coronavirus disease 2019 (COVID-19) caused by the severe acute respiratory syndrome coronavirus 2 (SARS-CoV-2) was first reported in December 2019, it has spread rapidly worldwide and has a huge impact on the public health and economies (1, 2). Unfortunately, specific antiviral therapies against SARS-CoV-2 are not available, making it difficult to achieve successful treatment for all COVID-19 patients. Some patients even experience rapid deterioration from onset of symptoms into severe cases with acute respiratory distress syndrome (ARDS) and other serious complications (3). Therefore, the early prediction and discrimination of severe COVID-19 cases versus moderate ones will not only alleviate the shortage of medical resources but also facilitate timely, appropriate supportive care to reduce the mortality rate.

Several risk factors, including diabetes, hypertension, primary cardiovascular disease, smoking history, gender, and age, have been found to be closely associated with the severity of COVID-19 (4, 5). After infection with SARS-CoV-2, the immune inflammatory reactions change rapidly with the ongoing infection process and are responsible for viral elimination and further recovery from the infection (6). However, exacerbated immune and inflammatory response in disease progression may lead to more severe and lethal outcomes, indicating their potential for the prediction of severity outcomes (3). Recent studies have revealed that lymphopenia (LYM) (7, 8), higher levels of serum proinflammatory cytokines (9), and higher total antibody titer (10) are the common features in most severe COVID-19 patients. Moreover, increased serum interleukin 6 (IL-6) level has also been reported to be associated with poorer prognosis and higher death rates in COVID-19 patients (4, 11). A prediction model consisting of IL-8, CD4^+^ T cell count, and NK cell count was reported to have good performance in predicting the prognosis of COVID-19 patients (12). Another report showed that age, LYM count, albumin (ALB) level, and neutrophil-to-lymphocyte ratio (NLR) were the independent high-risk factors for the severity of SARS-CoV-2 infection. The risk model based on these factors can effectively predict severe cases (13). Thus, several immune profiles, including cytokines, circulating cells, and inflammatory markers, are significantly changed in severe COVID-19 and have the potential to be used as predictors for severity of this disease.

Based on the dramatic contribution of immune reaction to the development of the disease, it is of great significance to explore the immune-related parameters to discriminate moderate from severe cases and provide different therapeutic strategies for them. However, prediction models based on immune reaction-related parameters for COVID-19 patients are rare. Therefore, there is still an urgent need for an accurate predictive immune model for clinical prediction as well as for scientific research. In this study, a prediction model based on immune and inflammatory response was constructed and applied for early prediction of severe cases in patients infected with SARS-CoV-2.

## RESULTS

### Screening of candidate predictors for COVID-19 patients.

**FIG 1:**
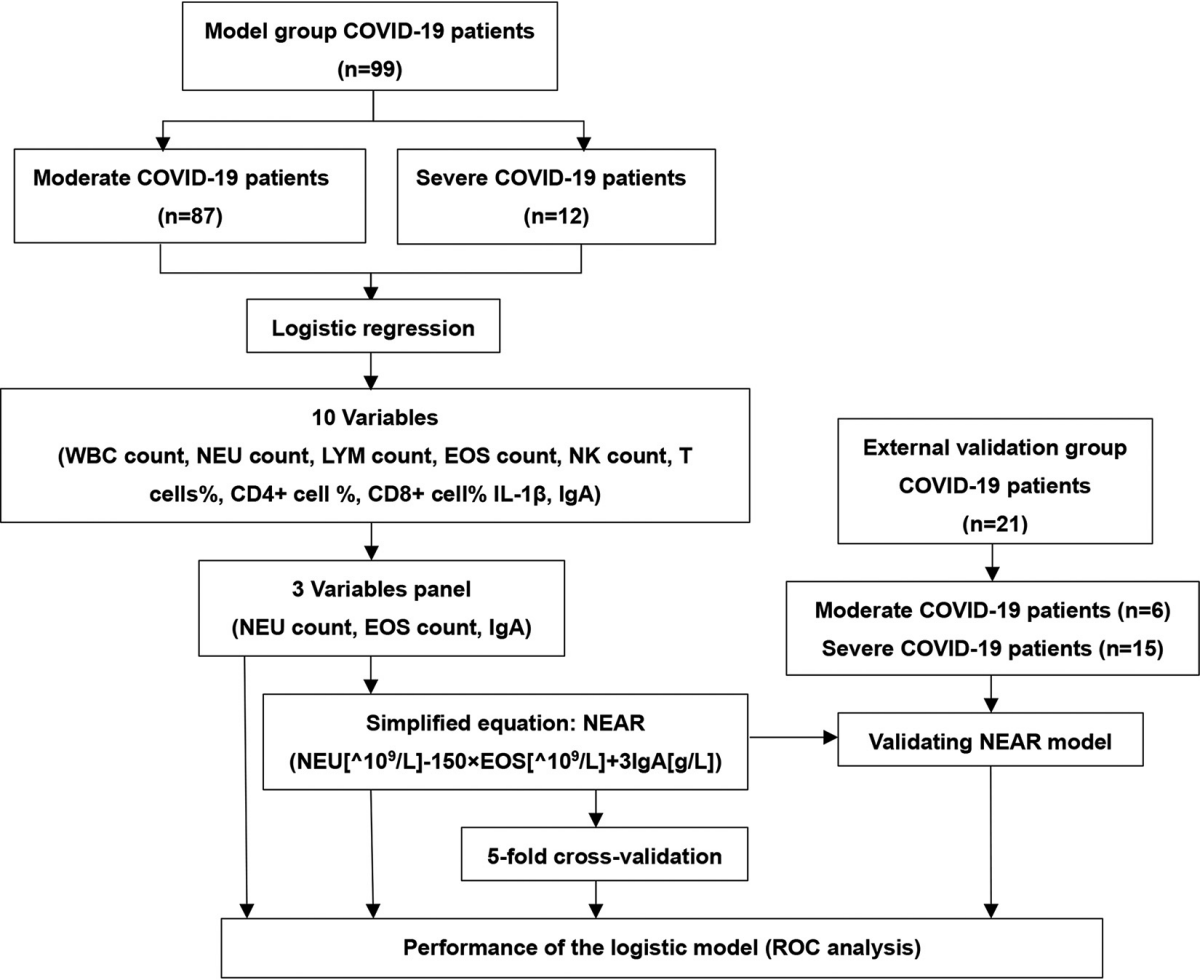
Flow chart of the study design and predictive model construction. ROC, receiver operating characteristic.

According to the grouping criteria mentioned above, all patients were classified in either the moderate-disease or the severe-disease group (Fig. 1). In brief, a group of symptoms, including shortness of breath, oxygen saturation, oxygen partial pressure, oxygen concentration, etc., was used to designate the severe cases. The demographic and immune response parameters are summarized in Table 1. Generally speaking, patients with severe disease were older than moderate-disease patients and included a moderately higher percentage of males than females; however, these data did not show statistically significant differences, probably due to the limited number of recruited patients. In term of circulating blood cells, the counts of white blood cells (WBC) and neutrophils (NEU) in severe patients were significantly increased, while the counts of LYM and eosinophils (EOS) were significantly decreased. The quantities of lymphocyte subsets, including B cells, NK cells, CD8^+^ T cells, and CD4^+^ T cells, were investigated in both groups, and the data showed that T cell proportion, NK count, and CD4^+^ T cell proportion in severe patients were all significantly decreased, whereas CD8^+^ T cell proportion was significantly elevated. With regard to serum cytokine profiles and immune-related parameters, including globulin, IL-1β, IL-2, IL-12, and IgA, their values in severe-disease patients were significantly higher than in those with moderate disease. Moreover, the severe-disease patients showed a significantly reduced ALB level compared with the moderate-disease patients. No significance difference was seen in complement (C3 and C4) level, B cell count and proportion, or the proportion of CD4^+^ and CD8^+^ T cells, IL-4, IL-5, IL-6, IL-8, IL-10, IL-17, tumor necrosis factor alpha (TNF-α), interferon (IFN), etc. These data indicated that there were significant differences associated with the immune responses to severe and moderate disease, which indicated a promising strategy for screening the valuable parameters for predicting the severe patients.

**TABLE 1:** Characteristics of the model group patients with COVID-19^*a*^

Characteristics	Value for group with COVID-19 severity		*P* ^*b*^
	Moderate (*n* = 87)	Severe (*n* = 12)	
Gender			
Male, n (%)	48 (48.0)	8 (8.0)	
Female, n (%)	39 (39.0)	4 (4.0)	0.544
Age (yrs)	42 ± 19	55 ± 22	0.0900
WBC count (10^9^/liter)	5.33 (4.11, 6.46)	7.14 (5.54, 9.92)	0.0009*
NEU proportion (%)	52.62 ± 17.46	68.45 ± 4.31	0.2186
LYM proportion (%)	39.46 ± 17.27	21.80 ± 3.82	0.1667
MON proportion (%)	6.31 ± 2.62	9.45 ± 0.35	0.1073
NEU cells count (10^9^/liter)	3.00 (2.30, 3.89)	5.86 (4.56, 8.59)	<0.0001*
LYM cells count (10^9^/liter)	1.63 (1.10, 2.00)	0.68 (0.37, 1.27)	0.0009*
MON cells count (10^9^/liter)	0.40 (0.30, 0.49)	0.42 (0.23, 0.61)	0.7726
EOS cells count (10^9^/liter)	0.05 (0.02, 0.09)	0.00 (0.00, 0.01)	<0.0001*
BAS cells count (10^9^/liter)	0.02 (0.01, 0.02)	0.01 (0.01, 0.02)	0.1996
T cell proportion (%)	66.55 (56.83, 71.84)	47.65 (42.25, 65.40)	0.0033*
NK cell proportion (%)	32.50 (22.90, 36.90)	26.40 (23.05, 32.60)	0.3329
NK cell count (10^3^/ml)	22.60 (17.40, 28.80)	15.70 (11.05, 20.25)	0.0044*
B cell proportion (%)	1.57 (1.12, 2.50)	1.62 (1.36, 2.36)	0.7305
B cell count (10^3^/ml)	14.40 (8.50, 23.80)	17.20 (8.93, 27.58)	0.6174
CD4^+^ cell proportion (%)	40.42 ± 8.25	15.76 ± 11.99	0.0008*
CD8^+^ cell proportion (%)	12.20 (8.50, 21.46)	20.80 (16.53, 41.30)	0.0051*
CD4^+^/CD8^+^	1.53 ± 0.55	0.35 ± 0.33	0.0095*
CD4^+^ cell count (10^3^/ml)	938.08 ± 385.64	325.50 ± 238.29	0.0540
CD8^+^ cell count (10^3^/ml)	668.17 ± 259.51	1089.50 ± 335.88	0.0609
IL-1β (pg/ml)	16.71 (5.10, 26.85)	77.53 (53.90, 161.70)	0.0019*
IL-2 (pg/ml)	2.93 (2.69, 3.12)	3.97 (3.23, 9.25)	0.0092*
IL-4 (pg/ml)	2.92 (2.48, 3.39)	3.45 (3.17, 6.06)	0.0785
IL-5 (pg/ml)	3.63 (3.05, 4.42)	4.09 (2.38, 5.81)	0.5375
IL-6 (pg/ml)	2.60 (2.44, 19.91)	3.81 (2.44, 4.63)	0.8106
IL-8 (pg/ml)	16.92 (13.92, 35.73)	54.47 (34.51, 298.56)	0.1505
IL-10 (pg/ml)	2.44 (2.44, 2.74)	3.81 (2.44, 4.63)	0.0803
IL-12 (pg/ml)	3.11 (2.89, 3.39)	3.79 (3.31, 5.66)	0.0393*
IL-17 (pg/ml)	2.84 (2.52, 3.15)	3.29 (2.91, 4.32)	0.0632
TNF-α (pg/ml)	7.92 (2.69, 15.70)	7.73 (6.16, 21.44)	0.5525
IFN-α (pg/ml)	2.85 (2.50, 3.21)	3.65 (2.89, 4.49)	0.0800
IFN-γ (pg/ml)	4.34 (3.19, 12.70)	6.81 (4.98, 16.86)	0.1505
C3 (g/liter)	1.26 ± 0.29	1.18 ± 0.27	0.3829
C4 (g/liter)	0.27 (0.23, 0.32)	0.28 (0.22, 0.32)	0.8896
IgG (g/liter)	11.63 (10.40, 13.74)	13.33 (10.00, 15.51)	0.3622
IgA (g/liter)	1.78 (1.44, 2.34)	2.47 (1.88, 3.38)	0.0121*
IgM (g/liter)	1.01 (0.72, 1.38)	0.94 (0.76, 2.17)	0.9797
TP (g/liter)	66.40 (62.35, 71.20)	67.05 (57.75, 72.18)	0.7219
ALB (g/liter)	40.13 ± 4.77	36.49 ± 5.53	0.0170*
GLOB (g/liter)	26.00 (24.00, 29.00)	28.25 (22.25, 30.83)	0.4571
ALB/GLOB	1.50 (1.40, 1.70)	1.45 (1.20, 1.58)	0.0957

a Continuous variables are expressed as means ± SD for normal data or medians and interquartile ranges for nonnormal data. Comparison tests were performed using a *t* test or Mann-Whitney rank sum test as appropriate. Categorical variables are expressed as number (percent) and were compared by chi-square or Fisher exact tests.

b *, *P *< 0.05.

### Assessment of the predictive efficiency of the candidate predictors.

**FIG 2:**
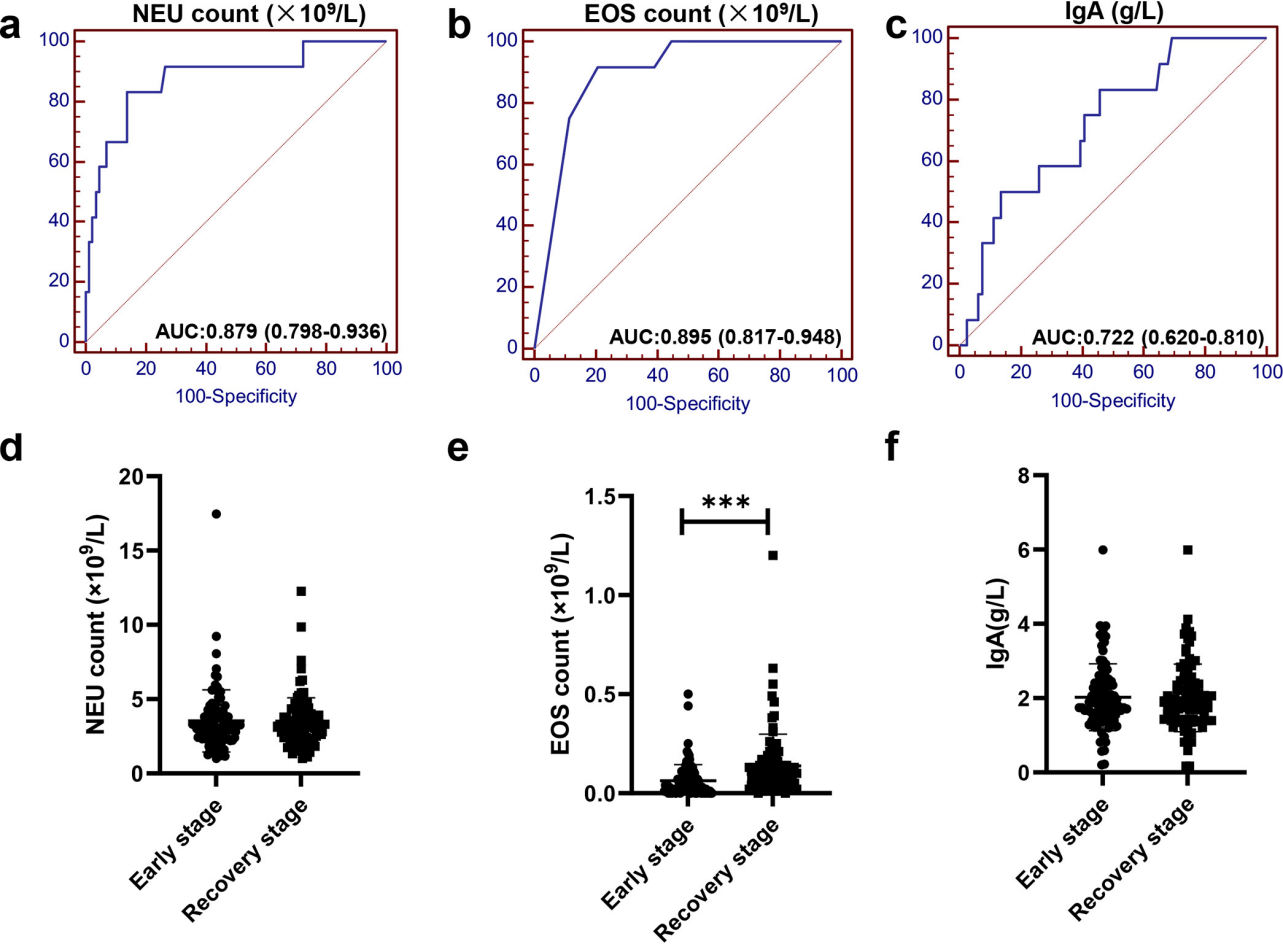
ROC curve analysis for prediction of severe COVID-19. AUC for NEU count (a), EOS count (b), and IgA level (c) for predicting severity of COVID-19; scatterplots showing NEU counts (d), EOS counts (e), and IgA level (f) in the early and recovery stages. ***, *P < *0.001 (paired *t* test).

These variables with significant differences between the severe and moderate cases were further screened as valuable candidate predictors. For the first step, all variables were analyzed by a univariate logistic analysis and were further assessed by binary logistic regression analysis. Thus, a total of 10 variables (WBC count, NEU count, LYM count, EOS count, NK count, ALB, T cells, CD8^+^ T cell proportion, IL-1β, and IgA) were identified as statistically significant parameters from the original variables using univariate analysis. IL-1β was discarded due to lack of data for some patients, and the remaining 9 variables were further assessed using a binary logistic regression assay. NEU count, EOS count, and IgA level were further screened and identified as independent predictors of severe COVID-19 (Table 2). These three indicators performed well to predict severity (Fig. 2a to c), and the best AUC among them was obtained from EOS count (Table 3 and Fig. 2b). Notably, an EOS count of ≥0.02 × 10^9^/liter has a sensitivity of 91.67% and a specificity of 79.31%. Among these three variables, the EOS count displayed superior predictive efficacy compared with the other two predictors. The EOS count of the patients in the early stage was significantly lower than that in the recovery stage, whereas the NEU count and IgA level display no significant difference between these stages (Fig. 2d to f). Thus, the significance of EOS count in the prediction can also be supported by the rapid restoration of the EOS count in the recovery stage (Fig. 2e). Together, these data indicated that the three indicators we identified have a good predictive capability and that EOS count, which can be efficiently obtained from acute COVID-19 infection, has unique predictive power.

**TABLE 2:** Univariate and multivariate analysis of routine laboratory data used to obtain critical factors to build the model^*a*^

Variable	Univariate^*b*^			Multivariate^*c*^					
				β	SE	*P*	Exp(β)	95% CI for Exp(β)	
	β	SE	*P*					Lower	Upper
WBC count (10^9^/liter)	0.487	0.167	0.004*						
NEU count (10^9^/liter)	0.758	0.216	0.0001*	0.724	0.264	0.006	2.063	1.230	3.459
LYM count (10^9^/liter)	−1.944	0.65	0.003*						
EOS count (10^9^/liter)	−93.03	34.57	0.007*	−130.457	59.881	0.029	0	0.000	0.000
EOS proportion (%)	−15.794	10.977	0.15						
NK count (10^3^/ml)	−0.147	0.063	0.019*						
ALB (g/liter)	−0.149	0.065	0.022*						
T cell proportion (%)	−0.094	0.03	0.002*						
CD4**^+^** T cell proportion (%)	−0.372	0.314	0.237						
CD8**^+^** T cell proportion (%)	0.088	0.029	0.003*						
CD4^+^/CD8^+^	−1,284.949	43,623.923	0.977						
IL-1β (pg/ml)	0.05	0.023	0.03*						
IL-2 (pg/ml)	1.511	0.88	0.086						
IL-12 (pg/ml)	1.462	0.803	0.069						
IgA (g/liter)	0.693	0.321	0.031*	2.562	1.229	0.037	12.964	1.167	144.030

a IL-1β was discarded due to lack of data from some patients.

b Univariate analysis of routine laboratory data to obtain meaningful factors.

c Further analysis to obtain more critical factors to build the model.

**TABLE 3:** Performance of various methods for distinguishing between severe and moderate disease

Variable	Cutoff value	Value (95% CI)			Accuracy (%)
		AUC	Sensitivity (%)	Specificity (%)	
WBC count (10^9^/liter)	5.36	0.787 (0.693–0.864)	91.67 (61.5–99.8)	51.16 (40.1–62.1)	56.12
NEU count (10^9^/liter)	4.36	0.879 (0.798–0.936)	83.33 (51.6–97.9)	86.21 (77.1–92.7)	85.86
LYM count (10^9^/liter)	1.02	0.785 (0.692–0.862)	75.00 (42.8–94.5)	80.46 (70.6–88.2)	79.80
EOS count (10^9^/liter)	0.02	0.895 (0.817–0.948)	91.67 (61.5–99.8)	79.31 (69.3–87.3)	80.81
NK count (10^3^/ml)	20.7	0.775 (0.659–0.867)	90.00 (55.5–99.7)	61.02 (47.4–73.5)	65.22
ALB (g/liter)	36	0.677 (0.575–0.768)	50.00 (21.1–78.9)	86.05 (76.9–92.6)	81.63
T cells (%)	50.8	0.756 (0.659–0.837)	58.33 (27.7–84.8)	90.70 (82.5–95.9)	86.73
CD8^+^ T cells (%)	16.1	0.747 (0.645–0.832)	83.33 (51.6–97.9)	62.03 (50.4–72.7)	64.84
IgA (g/liter)	1.86	0.722 (0.620–0.810)	83.33 (51.6–97.9)	54.32 (42.9–65.4)	58.06
NLR	3.72	0.876 (0.795–0.934)	83.33 (51.6–97.9)	88.51 (79.9–94.3)	87.88
PLR	186.25	0.762 (0.666–0.842)	83.33 (51.6–97.9)	70.11 (59.4–79.5)	71.71
MLR	0.38	0.799 (0.706–0.837)	75.00 (42.8–94.5)	82.76 (73.2–90.0)	81.82
SII	721.63	0.864 (0.780–0.925)	91.67 (61.5–99.8)	81.61 (71.9–89.1)	82.83
NEAR	9	0.961 (0.899–0.990)	100.00 (73.5–100.0)	88.89 (80.0–94.8)	90.32

### NEAR as a novel coefficient for predicting the severity of COVID-19.

**FIG 3:**
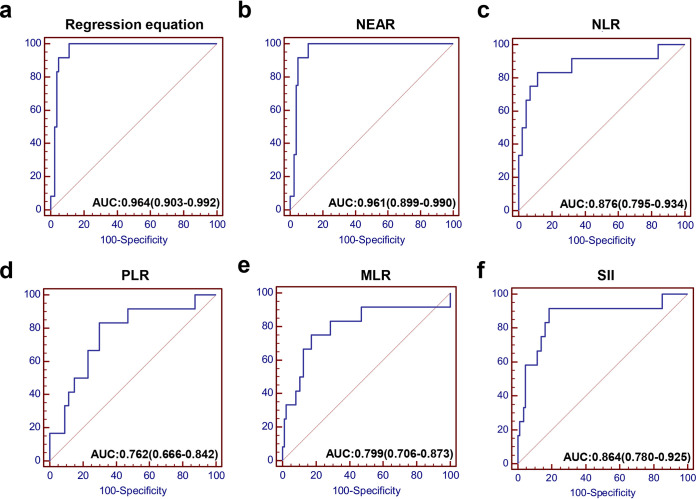
ROC curve analysis of NEAR and some models for prediction of severe COVID-19 patients. AUC of the regression equation (a), NEAR (b), NLR (c), PLR (d), MLR (e), and SII (f) predicting severity of COVID-19.

**FIG 4:**
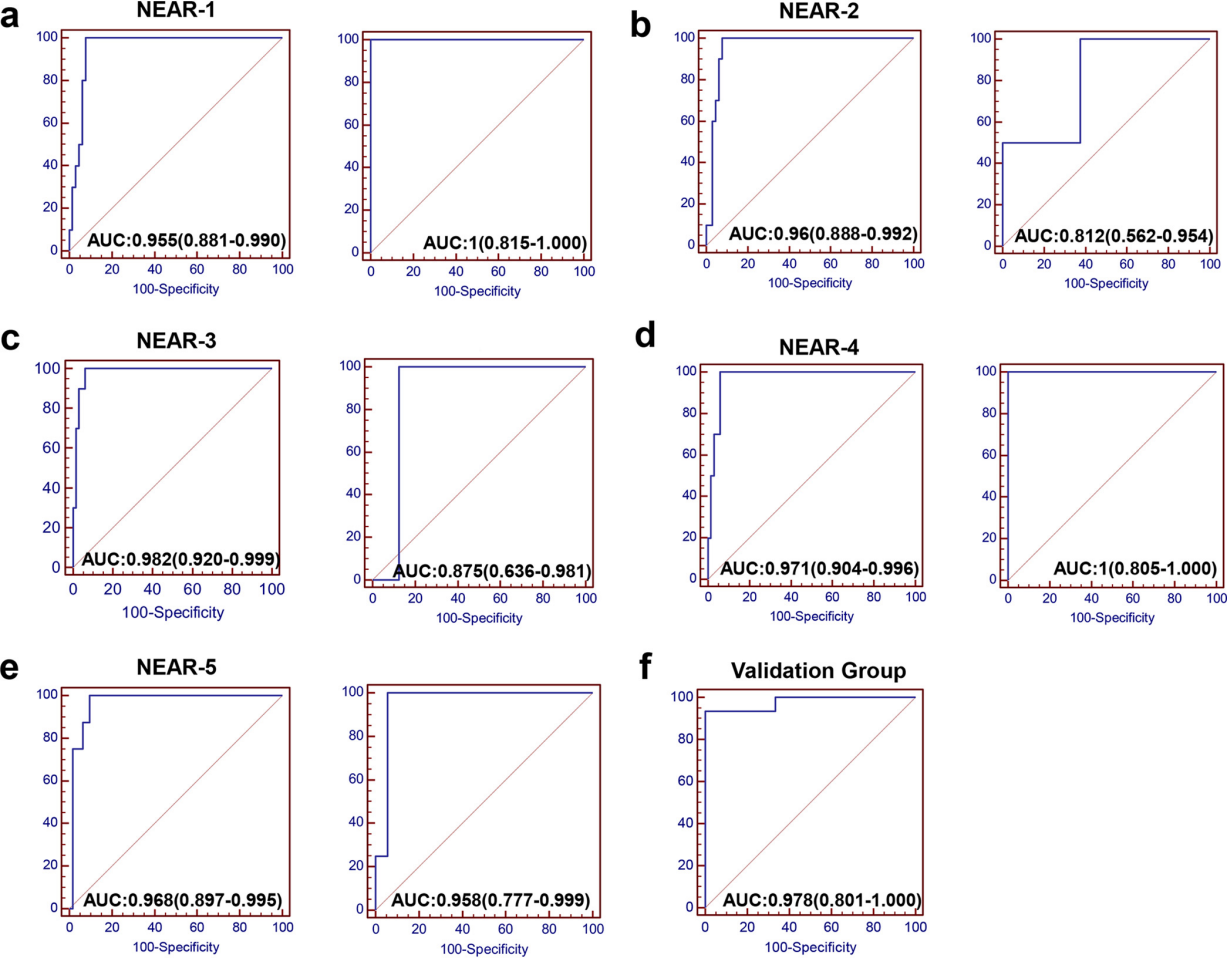
Validation of the NEAR model. A fivefold cross-validation approach was used to validate the predictive model NEAR (a to e). AUC of NEAR predicting severity of COVID-19 in (a) the first training set (left) and validation set (right), (b) the second training set (left) and validation set (right), (c) the third training set (left) and validation set (right), (d) the fourth training set (left) and validation set (right), and (e) the fifth training set (left) and validation set (right). (f) AUC of NEAR predicting severe COVID-19 in the validation group.

Using these three independent predictors identified by the binary logistic regression analysis, we constructed the prediction model as following: 0.724×NEU [10^9^/liter] − 130.457×EOS [10^9^/liter] + 2.562×IgA [g/liter] − 9.216, and the AUC value was 0.964 (Fig. 3a). However, the regression equation was complex and difficult to calculate; therefore, we simplified it into a new applicable equation: NEU[×10^9^/liter] − 150×EOS[×10^9^/liter] + 3×IgA[g/liter], and designated it NEAR (neutrophil, eosinophil, and IgA ratio). The AUC value of NEAR for predicting the severe patients was 0.961 (Fig. 3b), indicating that the regression equation and NEAR value had similar accuracy. The predictive performance of NLR, platelet-to-lymphocyte ratio (PLR), monocyte-to-lymphocyte ratio (MLR), and systemic immune inflammation index (SII) were also evaluated in this study (Fig. 3c to f), but none of them exhibited better predictive capability than our proposed model. We also use 5-fold cross-validation for further validation of the predictive efficiency of NEAR (Fig. 4a to e). NEU count, EOS count, and IgA level, which constituted the five-value prediction model, had an average AUC of 0.929. Although this average AUC is slightly lower than the above NEAR value, it still supported NEAR as a good prediction model for severe disease. We also verified NEAR in another cohort with IgA specific to the SARS-COV-2 antigen (Table S1) and found that the model also has a good predictive effect for severe disease (Fig. 4f).

10.1128/mSphere.00752-21.1 TABLE S1 Characteristics of the validation group patients with COVID-19. *, *P* < 0.05. Continuous variables are expressed as means and SD for normal data or medians and interquartile ranges for nonnormal data. Comparison tests were performed using a *t* test or Mann-Whitney rank sum test as appropriate. Categorical variables are expressed as number (percent) and were compared by chi-square or Fisher exact tests. Download Table S1, DOCX file, 0.02 MB. Copyright © 2021 Sun et al. 2021 Sun et al. https://creativecommons.org/licenses/by/4.0/ This content is distributed under the terms of the Creative Commons Attribution 4.0 International license.

Based on the receiver operating characteristic (ROC) curves of NEAR, the optimized cutoff value for prediction was set as 9 to distinguish severe from moderate cases (Table 3). Using the cutoff value of 9, 100% of COVID-19 patients with NEAR scores of >9 were verified as having severe disease, with good sensitivity and no missing cases. We also obtained the model’s positive predictive value (PPV) value of 57.14% and negative predictive value (NPV) of 100%. Hence, NEAR can distinguish severe COVID-19 cases from moderate cases with high-efficiency, and only a very small fraction of moderate cases were incorrectly included among severe cases, which can be corrected with a follow-up assay.

### NEAR had good performance in discriminating severe cases with different ages and gender.

**FIG 5:**
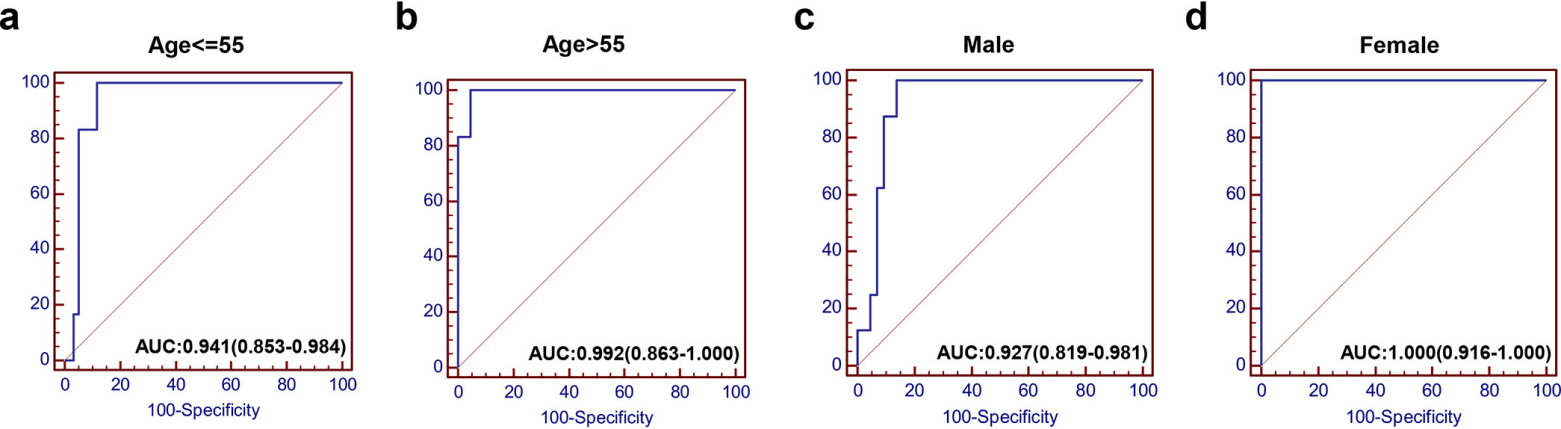
ROC curve analysis for prediction of severe COVID-19 patients in different age and gender subgroups. AUC of NEAR predicting severe COVID-19 in the young group (≤55 years, *n* = 70) (a) and the older group (>55 years, *n* = 29) (b); AUC of NEAR predicting severe COVID-19 in males (*n* = 56) (c) and females (*n* = 43) (d).

These COVID-19 patients were further grouped by average age (55 years) and gender, and the predictive efficacy of NEAR was further evaluated using the ROC assay in those groups. The results showed that AUC values were 0.992 (95% confidence interval [CI], 0.863 to 1.000) (Fig. 5a) for patients older than 55, 0.941 (95% CI, 0.853 to 0.984) (Fig. 5b) for patients no more than 55 years old, 0.972 (95% CI, 0.819 to 0.981) (Fig. 5c) for male patients, and 1.000 (95% CI, 0.961 to 1.000) (Fig. 5d) for female patients, respectively. Altogether, these data indicated that NEAR had a high predictive efficacy for all the recruited patients regardless of age and gender, and it had a broad range of clinical application.

### The parameters of NEAR were correlated with disease progression.

**FIG 6:**
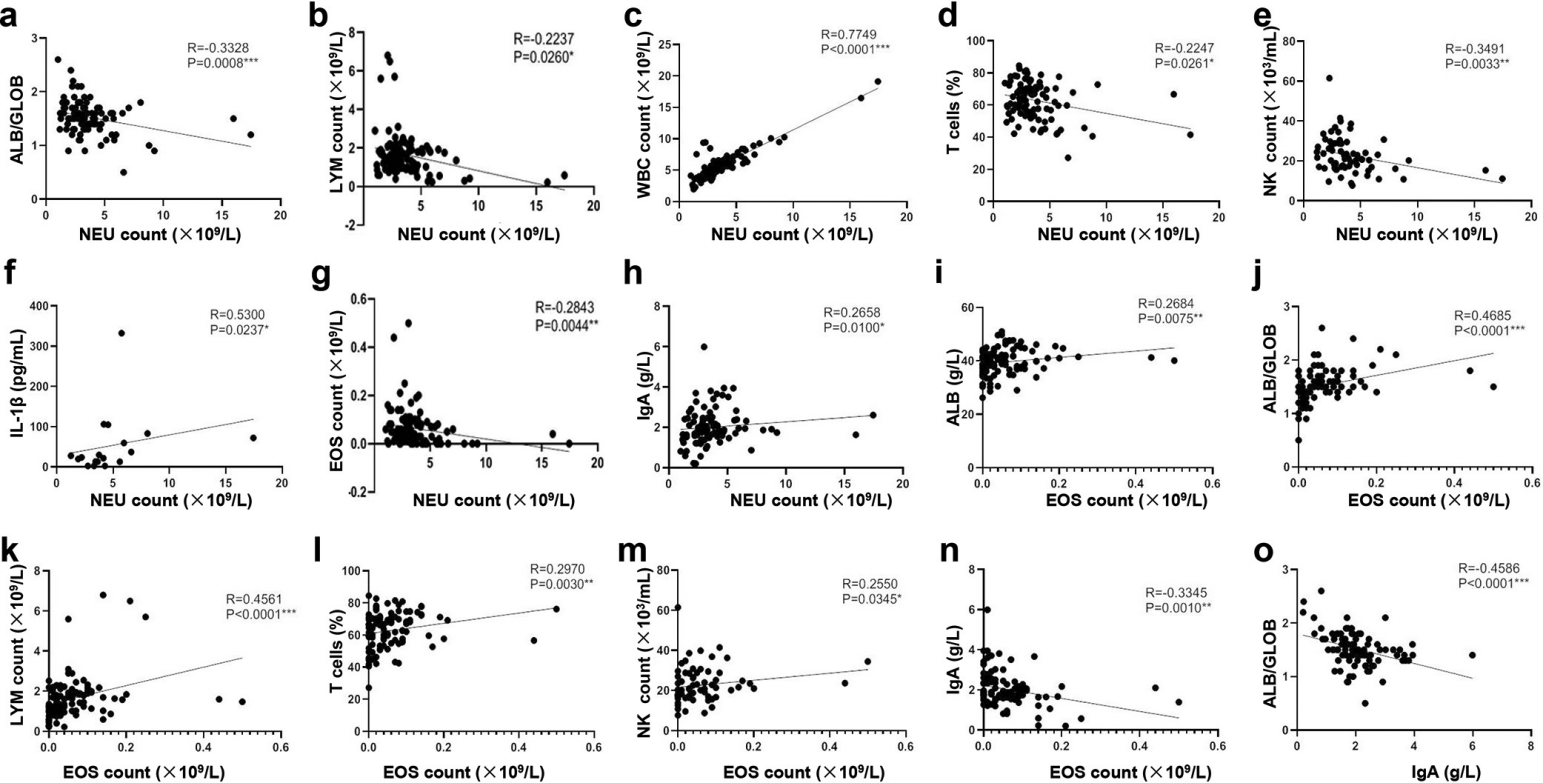
Spearman correlation analysis to verify the correlation between major indicators. Spearman correlation analysis of ALB/GLOB versus NEU count (a), LYM count versus NEU count (b), WBC count versus NEU count (c), T cells proportion versus NEU count (d), NK count versus NEU count (e), IL-1β versus NEU count (f), EOS count versus NEU count (g), IgA versus NEU count (h), ALB versus EOS count (i), ALB/GLOB versus EOS count (j), LYM count versus EOS count (k), T cell proportion versus EOS count (l), NK count versus EOS count (m), IgA versus EOS count (n), and ALB/GLOB versus IgA (o).

Using the Spearman correlation analysis, we further detected the correlation between these three indicators of the NEAR and other indicators associated with immune and inflammatory response. Our data showed that NEU count, EOS count, and IgA level were related to most other reported severity indicators, including the albumin-to-globulin ratio (ALB/GLOB), LYM count, WBC count, T cell proportion, NK cell proportion, IL-1β level, and IgA level, which further proved the essential role of these three selected indicators (Fig. 6a to o). Notably, NEU count was negatively correlated with EOS count (*R* = −0.2843; *P = *0.0044) (Fig. 6g) and positively correlated with IgA level (*R* = 0.2658, *P = *0.01) (Fig. 6h), while EOS count was negatively correlated with IgA level (*R* = −0.3345; *P = *0.001) (Fig. 6n). All these data tendencies were also consistent with the correlation tendency in the NEAR model. Our data further showed that the three indicators selected had good correlation with many immune indicators (Fig. 6a to o), which indicated that they might have a more comprehensive predictive ability and research potential.

## DISCUSSION

Since the outbreak of COVID-19, the rapidly increasing number of patients has exerted a high pressure on medical health service systems in regions undergoing the pandemic. Effective triage, hierarchical medical systems, and timely supplementation of medical resources play a crucial role in reducing the mortality of COVID-19. Therefore, there is an urgent need for an effective strategy to help clinicians accurately distinguish severe cases from moderate cases at an early stage of the disease. Here, we constructed a novel predictive model designated NEAR, which has a high accuracy for predicting severe COVID-19 with sensitivity and specificity of 100% and 88.89%, respectively.

In this study, among all of the indicators in the NEAR model, EOS count was found to have the highest individual predictive power for severe disease. Furthermore, compared to the decreased level in patients at the early stage of disease, EOS count was significantly restored in patients at the recovery stage, which indicated that EOS count was closely correlated with disease progression, indicating that it can be used as a new parameter for effectively monitoring the progression of and recovery from COVID-19. Our results were also supported by a recent study which showed that EOS count had a significantly decrease in most COVID-19 patients and could be used as an effective indicator for diagnosis, evaluation, and prognosis monitoring of COVID-19 patients (14). The reason for the decreased amount of circulating EOS in severe COVID-19 patients still remained to be clarified. Recent reports suggested that during virus infection and lung injury, circulating EOS are recruited to the lung tissue to mediate the antiviral response, and this might lead to the decreased amount of EOS in the circulatory system (15, 16). Other studies also showed that EOS were recruited to the lungs during the development of asthma (17). On the other hand, viral infection caused disturbance of the homeostasis of bone marrow, which might further lead to aberrant hematopoiesis (18) and abnormal production of EOS (19). Although several studies have explained the cause of downregulation of EOS in severe COVID-19 patients, the involved molecular mechanism has not been clarified and deserves further scientific investigation. The pivotal role of EOS in predicting severe cases in COVID-19 patients had a great significance for clinical validation and application.

Our study identified the abnormal level of serum IgA as a predictor for severe cases. As a mucosa-targeting virus, SARS-CoV-2 can induce the immune system to produce secretory IgA (sIgA) and induces strong antiviral mucosal immunity in the respiratory tract. Actually, mucosal antiviral immunity prevents pathogens from adhering to the cell surface through IgA-mediated interactions with pathogenic microorganisms (20). However, some reports have shown that IgA production against the SARS-CoV-2 spike protein appears early in infected patients and is closely related to the severity of COVID-19 (21–24). Xue et al. (25) also reported that the combination of IgA and IgG, which might prevent the infection and invasion of SARS-CoV-2, could actually further predict the progression of pulmonary lesions in severe COVID-19 patients. In line with these reports, we demonstrated that the serum IgA level was significantly upregulated in patients with severe disease and that this high level of serum IgA also had a relatively high predictive power. During the antiviral immune response, cross-linking of FcαRI by serum IgA can transmit activating signals, lead to respiratory burst, increase antigen presentation, and promote cytokine release (26). Cytokines such as transforming growth factor β (TGF-β) and IL-10 can further induce antibody isotype switching during this process to produce more IgA (27), and this seems to form positive feedback to boost inflammation. We used IgA as an indicator in NEAR, but in the process of external data verification, we found that replacing it with anti-SARS-COV-2 IgA also had good predictivity. This feature of our model has expanded its application, and both the total serum IgA level and the level of specific IgA against SARS-COV-2 could be used as the IgA value. Therefore, serum IgA level may be a valuable diagnostic marker to reflect the severe inflammation induced by virus infection, as a supplement to the well-recognized IgM/IgG detection (22). In addition, our study further indicated IgA as a promising predictor for severe disease, as presented here for the first time.

The individual predictive power of each indicator is relatively high (AUCs of NEU, EOS, and IgA were 0.879, 0.895, and 0.722, respectively). Using regression analysis, we combined these three indicators and constructed a linear equation (NEAR) with AUC of 0.961 (95% CI, 0.899 to 0.990). The coefficients of this linear equation, which was verified by the correlation between these three indicators, was also consistent with the actual impact of each indicator on the severity of COVID-19 (14). These data indicated that the predictive value of NEAR is scientific and authentic for discriminating severe cases. Some inflammatory parameters recognized to be involved in the COVID-19 progression, such as NLR, PLR, MLR, and SII (28–32), were also evaluated here for their predictive efficacy for the severity of COVID-19. However, besides sensitivity and specificity, the prediction efficacy of NEAR was significantly higher than those of these inflammatory parameters.

Patients with severe COVID-19 need to be identified on admission by routine clinical tests, and our newly developed NEAR meets this demand. The components of NEAR can be obtained in most routine laboratory tests. If the value of NEAR is higher than the threshold, advanced medical monitoring and support are recommended for the patient. Otherwise, symptomatic treatment in wards for milder disease, such as cabin hospitals, can be recommended. In addition, NEAR can be further recommended for monitoring the disease progression of the severe patients, although more evidence is needed to verify this application.

The present study has some limitations. Although a total of 120 COVID-19 patients from three medical centers were enrolled in this study, the number of patients was still relatively small, especially for the severe-COVID-19 patients. We have not been able to collect a larger set of external data to verify our model; therefore, we hope that more researchers can share clinical data to verify the effectiveness of the model before it is actually applied in the clinic. In addition, this model was developed with data from an Asian population, and it is of great scientific significance to verify the predictive efficiency of this model in other races and other centers outside Asia.

In conclusion, we constructed a predictive model and suggested a novel coefficient, called NEAR, for distinguishing severe from moderate COVID-19 cases. This model can be easily applied in clinical trials and help to discriminate patients with severe disease at an early stage, which may provide an opportunity for clinicians to optimize clinical treatment and rationally allocate limited medical resources.

## MATERIALS AND METHODS

### Ethics approval and consent to participate.

The study protocol was approved by Ethics Committee of Shandong Provincial Chest Hospital and Jinan Infectious Disease Hospital. Informed consent was obtained according to the committee’s principles. All methods were performed in accordance with the relevant guidelines and terms of the committee. This study was approved by the ethics Committee of First Affiliated Hospital of Guangzhou Medical University with approval number 2020-77.

### Participants.

The training model group included 99 COVID-19 patients in Shandong Provincial Chest Hospital and Jinan Infectious Disease Hospital recruited from January to May 2020. The validation group included 21 COVID-19 patients in the First Affiliated Hospital of Guangzhou Medical University recruited from February to April 2020. The suspected cases, which occurred in patients who had had exposure to the areas where the epidemic occurred and/or had typical clinical manifestation, such as fever and respiratory symptoms, were diagnosed with COVID-19 if the symptoms were accompanied by one of the following etiological or serological indicators: (i) real-time PCR (RT-PCR) detection of SARS-CoV-2 nucleic acid; (ii) viral gene sequence highly homologous to known SARS-CoV-2; or (iii) serum SARS-CoV-2-specific IgM and IgG, or conversion of serum IgG from negative to positive or a value four times or more higher in the recovery period than in the acute phase. The patients were diagnosed with COVID-19 infection, and severe cases were distinguished from moderate cases according to the 7th edition of the COVID-19 diagnosis and treatment protocol issued by the National Health Committee of the People’s Republic of China (http://www.nhc.gov.cn/) (33). Adults who met any of the following conditions were considered to have severe cases: (i) shortness of breath, with a respiration rate (RR) of ≥30 times/min; (ii) in the resting state, oxygen saturation of ≤93%; (iii) arterial partial pressure of oxygen (PaO_2_)/oxygen concentration (FiO_2_) of ≤300 mm Hg; (iv) lung imaging showing that the lesions progressed significantly within 24 to 48 h, i.e., >50%. Children who met any of the following conditions were considered to have severe cases: (i) shortness of breath (<2 months old, RR ≥ 60 times/min; 2 to 12 months old, RR ≥ 50 times/min; 1 to 5 years old, RR ≥ 40 times/min; >5 years old, RR ≥ 30 times/min), except for the effects of fever and crying; (ii) in the resting state, oxygen saturation of ≤92%; (iii) assisted respiration (groaning, flaring of alae nasi, three concave signs), cyanosis, or intermittent respiratory arrest; (iv) drowsiness or convulsions; (v) anti-feeding or feeding difficulties, with signs of dehydration. Respiratory failure, mechanical ventilation, shock, or other organ failures that require intensive care unit (ICU) monitoring and treatment are considered severe.

### Study design.

This study was designed to include a training group and a validation group. For the training group, because of the limited number of patients with mild disease (*n* = 6) in this retrospective study, the patients with mild and moderate disease were combined into one group for analysis. Therefore, the patients were divided into two groups: the moderate-disease group (*n* = 87) and the severe-disease group (*n* = 12). These two groups were used to train and validate the predictive model by using a 5-fold cross-validation method. For the validation group, the COVID-19 patients were confirmed and classified into moderate-disease (*n* = 6) and severe-disease (*n* = 15) groups and used as a model group for verification.

### Laboratory evaluation.

Laboratory confirmation of SARS-CoV-2 infection was performed using RT-PCR of nasopharyngeal swabs specimens following the protocol established by the World Health Organization (WHO) (34). For the sake of personal privacy, all patients’ personal information was hidden during the collection process. The following data were collected: demographic data, circulating blood cells, lymphocyte subset, serum cytokine profile, and immunoglobulin complement serum level. The circulating blood cell counts included the counts of WBC, NEU, LYM, monocytes (MON), EOS, and basophils (BAS) and the proportion of LYM, MON, and NEU; lymphocyte subset counts included the counts of NK cells, B cells, and CD4^+^ and CD8^+^ cells. The proportion of immune cells included those of T cells, B cells, and NK cells; immunoglobulin and complement serum levels included IgG, IgA, IgM, C3, and C4. Serum cytokine profiles and chemokines included IL-1, IL-2, IL-4, IL-5, IL-6, IL-8, IL-10, IL-12, IL-17, TNF-α, and IFN. We collected data from all patients upon admission and before discharge.

### Fivefold cross-validation.

We use R language to randomly divide the collected cases into 5 groups according to the proportion of moderate and severe cases. Then, we took 1 group each time as the validation group and the remaining 4 groups as the training group. For instance, where one group was used as the validation group and the rest as the training group, the cross-validation was done 5 times, and the average AUC value for the 5 times was used as the AUC of the 5-fold cross-validation (35).

### Statistical analysis.

Statistical analysis was carried out with SPSS software (version 22.0, SPSS Inc., USA) and GraphPad Prism software (version 9.0; GraphPad, La Jolla, CA). Continuous variables that conform to the normal distribution are presented as means and standard deviations (SD), and continuous variables that are not consistent are presented as medians and interquartile ranges (IQR). Categorical variables are reported as percentages. The difference between two groups (moderate versus severe) was analyzed by Student's *t* test or the Mann-Whitney rank sum test for continuous variables and by chi-square test for categorical variables. Correlation was performed by Spearman’s correlation analysis. Values were considered significant if *P* was <0.05. Univariate analysis was performed on all the demographic and laboratory variables by the log-rank test. We calculated a stepwise forward logistic regression with all significant variables to identify the independent risk factors and construct the predictive model based on immune profile for discrimination of severe COVID-19 cases. The predictive efficiency of the model was assessed by area under the curve in receiver operating characteristic curve analyses. The optimal cutoff values for diagnosis were selected using Youden’s index, which were maximal values at the sum of the sensitivity and specificity.

### Data availability.

All relevant data that support the findings of this study are available from the corresponding author upon request.
