# Supplementary material for: Early Prediction of Severe COVID-19 in Patients by a Novel Immune-Related Predictive Model
Source: mSphere. 2021 Oct 13;6(5):e00752-21. doi: 10.1128/mSphere.00752-21 (PMC8513681; doi:10.1128/mSphere.00752-21)
Supplement: TABLE S1 [file msphere.00752-21-st001.docx]

**Supplemental Material**

**Title: Early prediction of severe COVID-19 patients by a novel immune related predictive model**

**Supplementary Table S1** Characteristics of the validation group patients with COVID-19

| **Characteristics** | **Moderate (n = 6)** | **Severity (n = 15)** | ***P*** |
| --- | --- | --- | --- |
| Gender |  |  |  |
| Male, n (%) | 4 (19.0%) | 13 (61.9%) |  |
| Female, n (%) | 2 (9.5%) | 2 (9.5%) | 0.2920 |
| Age (years) | 55 ± 19 | 57 ± 14 | 0.7733 |
| WBC count (×10^9^/L) | 4.10 (3.28, 5.45) | 9.90 (7.50, 12.40) | 0.0023^*^ |
| NEU cells count (×10^9^/L) | 2.85 (1.93, 4.30) | 9.20 (7.00, 11.80) | 0.0011^*^ |
| LYM cells count (×10^9^/L) | 0.75 ± 0.23 | 0.40 ± 0.24 | 0.0071^*^ |
| EOS cells count (×10^9^/L) | 0.00 (0.00, 0.01) | 0.00 (0.00, 0.00) | 0.0714 |
| BAS cells count (×10^9^/L) | 0.00 (0.00, 0.00) | 0.00 (0.00, 0.00) | 0.5714 |
| T cells proportion (%) | 76.50 (72.00, 79.08) | 66.00 (59.80, 68.40) | 0.0017^*^ |
| NK cells proportion (%) | 9.97 ± 3.28 | 10.79 ± 7.79 | 0.8089 |
| NK cells count (×10^3^/mL) | 67.00 (65.00, 160.75) | 53.00 (9.00, 70.00) | 0.0910 |
| B cells proportion (%) | 12.40 (9.23, 16.60) | 25.45 (15.18, 31.60) | 0.0256^*^ |
| B cells count (×10^3^/mL) | 109.5 (82.75, 147.00) | 86.00 (67.00, 95.5) | 0.1860 |
| CD4^+^ cells proportion (%) | 47.70 ± 17.20 | 42.19 ± 13.96 | 0.5540 |
| CD8^+^ cells proportion (%) | 18.60 (13.90, 34.30) | 16.20 (6.90,32.80) | 0.5613 |
| CD4^+^/CD8^+^ | 3.02 (0.81, 4.26) | 2.56 (1.05, 5.26) | 0.6544 |
| CD4^+^ cells count (×10^3^/mL) | 449.00 (153.00, 516.00) | 116.00 (84.00, 308.00) | 0.0735 |
| CD8^+^ cells count (×10^3^/mL) | 158.67 ± 49.00 | 91.53 ± 82.78 | 0.1997 |
| IL-2 (U/L) | 0.82 (0.70, 1.27) | 1.22 (0.70, 1.96) | 0.4818 |
| IL-4 (U/L) | 1.61 ± 0.81 | 1.78 ± 0.69 | 0.6354 |
| IL-6 (U/L) | 12.61 (8.34, 35.45) | 26.87 (14.26, 86.05) | 0.1781 |
| IL-10 (U/L) | 2.95 (2.09, 4.69) | 5.52 (4.26, 9.27) | 0.0243^*^ |
| TNF-α (U/L) | 1.09 ± 0.36 | 1.50 ± 0.47 | 0.0707 |
| IFN-γ (U/L) | 0.95 (0.70, 3.38) | 1.32 (1.16, 1.72) | 0.2282 |
| anti-SARS-COV-2 IgG (mg/L) | 11.16 ± 15.60 | 41.18 ± 24.62 | 0.0127^*^ |
| anti-SARS-COV-2 IgA (mg/L) | 10.36 (7.23, 60.01) | 21.98 (5.54, 57.75) | 0.6897 |
| anti-SARS-COV-2 IgM (mg/L) | 2.44 (0.88, 3.80) | 2.80 (2.06, 5.16) | 0.3403 |
